# Supplementary material for: Chromothripsis during telomere crisis is independent of NHEJ, and consistent with a replicative origin
Source: Genome Res. 2019 May;29(5):737–49. doi: 10.1101/gr.240705.118 (PMC6499312; doi:10.1101/gr.240705.118)
Supplement: Supplemental Material [file supp_gr.240705.118_Supplemental_file_1.zip › contigs/annotated_contigs/DB78/contig.2.DB78_length_343_mean_cov_6.9970845481.docx]

**DB78_length_343_mean_cov_6.9970845481**

```
TGGAGCATTTGGGAAGCCTGAAGCCTGCCCTTCCTGGCAGCATCAGAAAGCAAGGAGAGATGTTGGTGTACAGGCAAAAGTCAGCTCTC
 >chr5:5556212-5556422 + E=4e-112
AACACAGGTGATGGAGGACTGTGTATGTAGCCAGGGATAAAGAATCCAGGAGGGGCCTGAAGATGGGTGGAACCAATGAAATGAATCAT

CCCAGGTGTATCATCTCTTGTTTGGAAATATC|AAGATGT|ATATATATATACAATGGAATACTACTCAGCCATAAAAAGGAATGAATT
                                        >chr5:5561282-5561408 + E=2e-58
ATGGTCCTTTAATGGGTATGAAAGTATATGTAAGTTTCAGGGTCTCTTTCCTCTGAGATGCCTCTGGTACTGATGGCT
```
